# Exosomal let-7d-3p and miR-30d-5p as diagnostic biomarkers for non-invasive screening of cervical cancer and its precursors

**DOI:** 10.1186/s12943-019-0999-x

**Published:** 2019-04-02

**Authors:** Mengyue Zheng, Ling Hou, Yu Ma, Lanyun Zhou, Fenfen Wang, Bei Cheng, Wei Wang, Bingjian Lu, Pengyuan Liu, Weiguo Lu, Yan Lu

**Affiliations:** 1 Center for Uterine Cancer Diagnosis & Therapy Research of Zhejiang Province, Women’s Reproductive Health Key Laboratory of Zhejiang Province, Department of Gynecologic Oncology, Women’s Hospital, Zhejiang University School of Medicine, Hangzhou, China; 2 Institute of Translational Medicine, Zhejiang University School of Medicine, Hangzhou, China; 3 Department of Clinical Laboratory, Tongde Hospital of Zhejiang Province, Hangzhou, China; 4 Department of Respiratory Medicine, Sir Run Run Shaw Hospital, Zhejiang University School of Medicine, Hangzhou, China; 5 Department of Physiology and Center of Systems Molecular Medicine, Medical College of Wisconsin, Milwaukee, WI USA; 6 Women’s Hospital and Institute of Translational Medicine, Zhejiang University School of Medicine, Zhejiang, 310029 Hangzhou China

**Keywords:** Cervical cancer, Diagnosis, Early detection, Exosome, miRNA, Next-generation sequencing, Liquid biopsy

## Abstract

Cervical cancer screening through detection and treatment of high-grade cervical intraepithelial neoplasia (CIN) is most successful in cancer prevention. However, the accuracy of the current cervical cancer screening tests is still low. The aim of this study was to develop a more accurate method based on circulating exosomal miRNAs. The miRNA sequencing was performed to identify candidate exosomal miRNAs as diagnostic biomarkers in 121 plasma samples from healthy volunteers, cervical carcinoma patients, and CIN patients. A panel with eight differentially expressed exosomal miRNAs was identified to distinguish patients in the CIN II+ group (including advanced CIN II patients) from those in the CIN I− group (including CIN I patients and healthy volunteers). Let-7d-3p and miR-30d-5p showed significant difference between cervical tumors and adjacent normal tissues (*P* < 0.005), exhibited a consistent trend in plasma samples, and were further validated in 203 independent plasma samples. Integrating these two miRNAs yielded an AUC value of 0.828 to distinguish patients in CIN II+ group from those in CIN I− group. Further integrating them into a cytological test-based model resulted in a higher AUC of 0.887, while the AUC value based on the cytological test alone was 0.766. In summary, plasma exosomal miR-30d-5p and let-7d-3p are valuable diagnostic biomarkers for non-invasive screening of cervical cancer and its precursors. Further validation using large sample sizes is required for clinical diagnosis.

**Electronic supplementary material:**

The online version of this article (10.1186/s12943-019-0999-x) contains supplementary material, which is available to authorized users.

Cervical cancer (CC) is the second leading cause of cancer death in women aged 20 to 39 years. Its crude incidence and mortality are 98.9 and 30.5 per 100,000, respectively, with an increasing trend in China [1]. CC screening is of great importance in identifying high-grade cervical intraepithelial neoplasia (CIN) in order to prevent their progression into invasive cancer. Screening tests such as the Papanicolaou test (Pap smear) and Thinprep Cytological Test (TCT) dramatically reduced the incidence of and increased the 5-year survival rate of cervical cancer [2]. However, the diagnosis rates of the Pap smear and TCT are still low. These cytological tests vary significantly in different regions and hospitals. They are not commonly used in all regions in China, especially in the rural areas. Most women take these tests when they have symptoms like abnormal vaginal bleeding, leucorrhea, abdominal pain, etc. Several factors restrict the extensive application of these tests, such as personal beliefs and cultural factors (especially in women older than 45 years or in rural areas), the risk of vaginal infection and bleeding, and the complexity and variability of the procedure.

Exosomes are 30–150 nm tiny vesicles found in all body fluids, and are one of the key subjects in liquid biopsy in precision medicine [3]. Exosomes deliver enriched genetic materials of DNA fragments, mRNA, long non-coding RNA, small RNA, proteins, and lipids, which are closely related to cancer development and progression [4]. Compared with the complex mechanisms of long non-coding RNA, heterogeneous mutation sites of cell-free DNA, and unstable characteristics of mRNA, exosomal miRNAs are stable and relatively non-degradable, with relatively mature detection methods, making them promising diagnostic biomarkers for complex diseases such as cancer [5]. Recent studies have shown that exosomal miRNAs have the potential to be efficient biomarkers for the screening, diagnosis, and monitoring of cancers. For instance, five-miRNA gene signature could differentiate indolent and aggressive forms of prostate cancer [6]. miR-122, miR-192, miR-17-5p, and miR-25-3p are respectively enriched in different cancer tissues and abundantly secreted into the culture media of tumor-derived exosomes [7]. Several miRNAs or miRNA panels from plasma or serum have shown their potentials as noninvasive biomarkers for cervical squamous cell carcinoma (SCC) before and after surgery [8] and for the early detection of non-small cell lung cancer [9].

In the present study, we carried out one of the largest plasma miRNA studies for cancer biomarker discovery. Exosomal miRNA sequencing was performed in 121 plasma samples from healthy volunteers, cervical carcinoma patients, and precancerous patients. Differentially expressed miRNAs (DEmiRs) were then validated in 46 new cervical tumors and their matched adjacent tissues using qRT-PCR. Furthermore, two of the DEmiRs (miR-30d-5p and let-7d-3p) were further validated in 203 independent plasma samples using droplet digital PCR (ddPCR), and it was confirmed that the combination of these two exosomal miRNAs is promising and effective for early detection of cervical cancer. The flow chart for the study design is illustrated in Additional file 1: Figure S1.

## Results and discussion

### Retrospective analyses of medical records of cervical cancer patients

We first performed retrospective analyses of medical records of cervical cancer patients to evaluate the accuracy of current cytology tests (Additional file 2: Supplementary Methods). A total of 456 of 608 patients had at least one TCT or Pap smear record, and 498 of the total patients had tissue biopsy results; 468 of 608 patients had an HPV test, of which 445 were HPV positive and 23 were HPV negative (Additional file 3: Figure S2A). The pathological stages of tissue specimens obtained from the operation were used as the diagnostic criteria for each patient. TCT or Pap smears that were negative for Intraepithelial Lesion or Malignancy (NILM), or Low-grade Squamous Intraepithelial Lesion (LSIL) were classified as true positive results for CIN I patients. High-grade Squamous Intraepithelial Lesion (HSIL) or Atypical Squamous Cell of Undetermined Significance (ASC-US) /Atypical Squamous Cell—cannot exclude high-grade squamous intraepithelial lesion (ASC-H) / Atypical Glandular Cells—not otherwise specified (AGC-NOS) were classified as true positive results for CIN II-III, adenocarcinoma (ACC), or squamous cell carcinoma (SCC) patients. Based on the generally accepted gold standard described above, the overall detection rate of the cytology tests in all the 465 patients with cytology results was approximately 68.86% (CIN I 67.65%, CIN II-III 65.57%, SCC 73.71%, and ACC 65.71%) (Additional file 3: Figure S2B). The overall detection rate of the biopsy tests in all the 498 patients with biopsy results was approximately 93.17% (CIN I 76.92%, CIN II-III 92.76%, SCC 96.52%, and ACC 94.59%) (Additional file 3: Figure S2C). Retrospective analyses demonstrated that the accuracy of current cytology tests is relatively low when compared with cervical biopsy, but there is still much room for improvement in CC screening.

### Identification of differentially expressed miRNAs in exosomes

To develop a more accurate screening method based on circulating exosomal miRNAs, miRNA sequencing was performed in 121 plasma samples from healthy volunteers, cervical carcinoma patients, and precancerous patients. The miRNA expression levels were quantified by Reads Per Million (RPM) mapped reads and then normalized with log2(RPM + 1), which is the commonly used method for miRNAs quantification and normalization [10]. Detailed methods regarding plasma exosomal miRNA sequencing analysis are provided in Additional file 2: Supplementary Methods.

Proper classification of the studied subjects was not only important for identifying DEmiRs, but also critical for developing powerful diagnostic biomarkers for CC screening. According to clinical guidance, CIN I patients have a reversible disease response and may return to normal, and thus do not have to be treated with surgery and medication. Therefore, CIN I patients and healthy volunteers were combined into one group named CIN I-. A high-grade CIN (i.e., CIN II-III) patients and CC patients (i.e., ACC and SCC) need treatment and were thus combined into another group named CIN II+. Our aim was to identify circulating exosomal DEmiRs as diagnostic biomarkers for screening CC and high-grade CIN. This grouping strategy increased sample sizes in each group and maximized the possibility of discovering the diagnostic miRNAs. The average age of all 608 studied subjects was 50 ± 24 years, and the average age of the CIN I- and the CIN II+ groups was 50 ± 27 and 50 ± 24 years, respectively; thus, there was no significant age difference between the groups of patients.

**Fig. 1:**
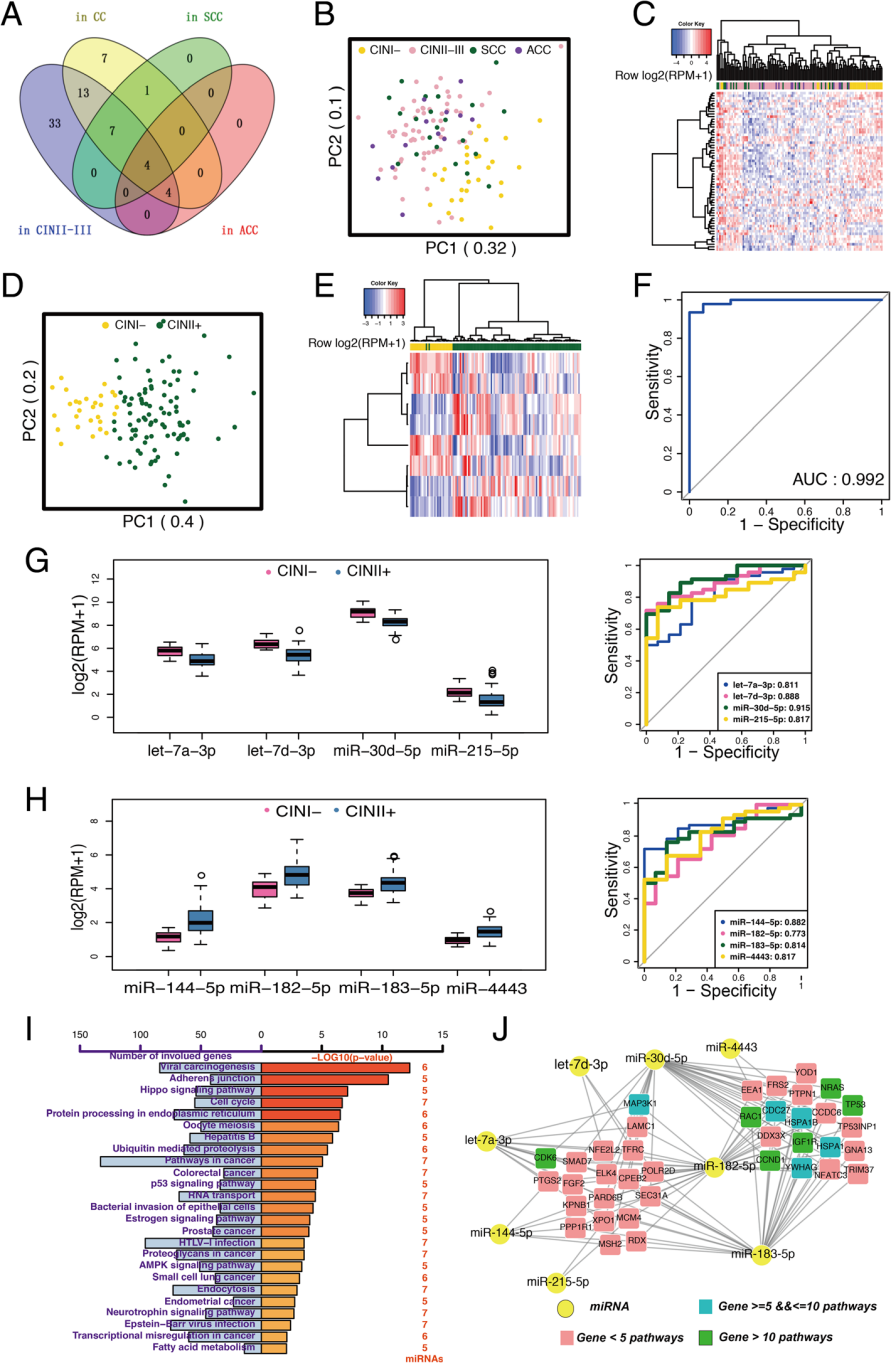
Identification of differentially expressed miRNAs in plasma exosomal sequencing samples. **a** Venn diagram of differentially expressed miRNAs between CIN I- and other groups (CIN II-III, CC, SCC, and ACC). **b**, **c** Principal component analysis (**b**) and clustering analysis (**c**) of all 61 significant exosomal miRNAs that were differentially expressed between CIN I- and other groups (CIN II-III, CC, SCC, and ACC). **d** ROC curves of the top eight significant miRNAs (let-7a-3p, let-7d-3p, miR-30d-5p, miR-144-5p, miR-182-5p, miR-183-5p, miR-215-5p, and miR-4443). ROC analysis was performed to evaluate the sensitivity and specificity of the eight-miRNA signature (i.e. a group of the top eight significant miRNAs) to discriminate CIN II+ from CIN I- subjects. **e**, **f** Principal component analysis (**e**) and clustering analysis (**f**) of the top eight significant miRNAs. **g**, **h** Expression levels and ROC curves of four down-regulated (**g**) and four up-regulated (**h**) miRNAs in CIN II+ group compared with those in CIN I- group. Exosomal miRNA expression levels were quantified as RPM in the sequencing data. **i** Biological pathways enriched for experimentally validated targets by at least five of the top eight miRNAs. Experimentally validated miRNA-target interactions were identified from the miRTarBase database. **j** miRNA-gene connection network. Circles represent miRNAs. Squares represent experimentally validated target genes by at least three of eight miRNAs. The pink, blue, and green squares represent target genes that were involved in < 5, 5–10, and > 10 significant pathways, respectively

A total of 312 miRNAs with mean log2(RPM + 1) values > 1 were detected from miRNA sequencing of exosomes derived from 121 plasma samples. Among these miRNAs, CIN I- samples were used as a reference data to compare with the other sample groups (CIN II-III, CC, SCC, and ACC). As a result, a total of 69 DEmiRs were identified in these four comparisons (false discovery rate, FDR < 0.01), of which 29 were identified in at least two comparisons (Table 1 and Fig. 1a). Specifically, 61 DEmiRs were identified between CIN I- and CIN II-III. Thirteen and eight DEmiRs were identified between CIN I- and SCC, and between CIN I- and ACC, respectively, of which four were common. Thirty-six DEmiRs were identified between CIN I- and CC and 28 were also identified between CIN I- and CIN II-III (Fig. 1a).

**Table 1:** List of significant miRNAs differentially expressed in at least two comparisons between CIN I- and other groups (CIN II-III, CC, SCC, and ACC)

Category^a^	CINII-III	CC	SCC	ACC
Set 1	let-7a-3p	let-7a-3p	let-7a-3p	let-7a-3p
	let-7d-3p	let-7d-3p	let-7d-3p	let-7d-3p
	miR-144-5p	miR-144-5p	miR-144-5p	miR-144-5p
	miR-30d-5p	miR-30d-5p	miR-30d-5p	miR-30d-5p
Set 2	miR-1468-5p	miR-1468-5p		miR-1468-5p
	miR-182-5p	miR-182-5p		miR-182-5p
	miR-215-5p	miR-215-5p		miR-215-5p
	miR-337-3p	miR-337-3p		miR-337-3p
	miR-10a-5p	miR-10a-5p	miR-10a-5p	
	miR-10b-5p	miR-10b-5p	miR-10b-5p	
	miR-148b-3p	miR-148b-3p	miR-148b-3p	
	miR-30a-5p	miR-30a-5p	miR-30a-5p	
	miR-409-5p	miR-409-5p	miR-409-5p	
	miR-4443	miR-4443	miR-4443	
	miR-96-5p	miR-96-5p	miR-96-5p	
Set 3		miR-425-5p	miR-425-5p	
	let-7b-3p	let-7b-3p		
	let-7e-5p	let-7e-5p		
	let-7f-1-3p	let-7f-1-3p		
	miR-145-3p	miR-145-3p		
	miR-183-5p	miR-183-5p		
	miR-193b-3p	miR-193b-3p		
	miR-214-5p	miR-214-5p		
	miR-27b-3p	miR-27b-3p		
	miR-365a-3p	miR-365a-3p		
	miR-483-5p	miR-483-5p		
	miR-574-3p	miR-574-3p		
	miR-656-3p	miR-656-3p		

^a^Differential expression levels of miRNAs were respectively examined in CIN I- vs. CIN II-III, CIN I- vs. CC, CIN I- vs. SCC, and CIN I- vs. ACC. Set 1 included miRNAs that were significant in four groups. Set 2 included miRNAs that were significant in three groups. Set 3 included miRNAs that were significant in two groups

Using all the DEmiRs detected above, principal component analysis (PCA) and clustering analysis were performed to assign these plasma samples into groups with similar miRNA expression patterns (Fig. 1b and c). Interestingly, CIN II-III and CC subjects shared common miRNA expression profiles. Furthermore, we also compared the expression of miRNAs between healthy and CIN I subjects in the discovery set, but none of the DEmiRs were found (FDR > 0.05). These results also justified our grouping strategy by which CIN II-III, ACC, and SCC patients were combined into one group, while healthy and CIN I subjects were combined into another group. Finally, the comparison of CIN I- with CIN II+ group identified 37 DEmiRs (FDR < 0.01), including 9 up-regulated and 28 down-regulated DEmiRs (Additional file 4: Table S1).

### Diagnostic accuracy of the exosomal miRNA panel to distinguish CIN I- and CIN II+ patients

Next, a set of miRNAs were selected from these 37 DEmiRs in the 121 plasma exosomal sequencing samples using the Random Forest algorithm. This led to the identification of the best panel with eight miRNAs (let-7a-3p, let-7d-3p, miR-30d-5p, miR-144-5p, miR-182-5p, miR-183-5p, miR-215-5p, and miR-4443) that are the strongest predictors in clinical diagnosis (i.e., CIN I- versus CIN II+). PCA was performed using these eight miRNAs; the first two principal components explained the 60% of total variance in the discovery set. They were visualized to show the groupings of these exosomal samples, indicating that samples in CIN I- and CIN II+ groups were nicely separated (Fig. 1d). Hierarchical clustering of these eight miRNAs indicated that only two CIN II+ patients were incorrectly classified into CIN I- group (Fig. 1e). ROC analysis was further performed to evaluate the sensitivity and specificity of the eight-miRNA signature to discriminate CIN II+ from CIN I- subjects. This yielded a very high AUC value of 0.992 (Fig. 1f). The AUC value of individual DEmiRs ranged from 0.797 to 0.890 in the discovery set (Fig. 1g and h). Furthermore, there were no significant differences in the expression of miRNAs in the best panel between different HPV types (*P* > 0.05). In summary, the newly identified eight-miRNA signature is highly predictive of CIN I- and CIN II+ irrespective of HPV types.

### Pathway enrichment analysis of diagnostic miRNAs

To gain further insight into the molecular function of these diagnostic miRNAs in CC, we performed enrichment analysis of Gene Ontology categories and Kyoto Encyclopedia of Genes and Genomes pathways on these miRNA targets (Additional file 2: Supplementary Methods). There were 25 significant pathways (FDR < 0.01) involved in at least five of the eight DEmiRs in the best panel (Fig. 1i) and most of them were cancer-related pathways, such as adherens junction, hippo signaling pathway, cell cycle, p53 signaling pathway, AMPK signaling pathway, and so on. Interestingly, the top targeted pathway was viral carcinogenesis, which was consistent with CC caused by HPV. Oocyte meiosis and estrogen signaling pathways were also significant. The connection network showed genes targeted by at least three miRNAs. Notably, miR-30d-5p, miR-182-5p, and miR-183-5p simultaneously regulate genes *RAC1, IGF1R, NRAS, TP53,* and *CCND1,* which were involved in more than 10 of the 25 significant pathways, and also regulate *CDC27* and *YWHAG,* which were involved in 5 to 10 of the 25 significant pathways. Furthermore, these eight miRNAs also regulated several other important cancer genes, including *CDK6*, which was involved in more than 10 pathways, and *MAP3K1*, which was involved in 5 to 10 pathways (Fig. 1j). These results demonstrated that the exosomal miRNAs detected in our sequencing study can not only serve as potential diagnostic biomarkers, but can also be identified as potential anti-cancer drug targets because they are functionally involved in the development and progression of CC.

### Validation of diagnostic miRNAs in tissues by qRT-PCR

We next used qRT-PCR to evaluate eight DEmiRs in the best panel for discriminating CIN I- from CIN II+ (Fig. 1g and h) in paired cancerous and para-carcinoma tissues from 46 new CC patients (Additional file 5: Figure S3A and B). Five miRNAs (let-7a-3p, let-7d-3p, miR-30d-5p, miR-183-5p, and miR-182-5p) showed consistent variation trends in plasma exosomes, among which three (let-7a-3p, let-7d-3p and miR-30d-5p) showed significant differences in expression between cancerous and para-carcinoma tissues. However, other three of the eight miRNAs (miR-215-5p, miR-144-5p, and miR-4443) showed either no changes or reversed trends in the tissues compared with plasma exosomes.

### Validation of diagnostic miRNAs in independent plasma samples by ddPCR

To validate these diagnostic miRNAs by ddPCR, four stably expressed miRNAs (i.e., miR-128-3p, miR-129-5p, miR-320a, and Let-7i-5p) were chosen from the exosomal miRNA sequencing data in the discovery set (Additional file 2: Supplementary Methods). These four miRNAs had relatively high expression levels (log2(PRM + 1) > 10) and small variability among samples (coefficient of variation < 5%) (Additional file 6: Figure S4). They were used as endogenous references for normalizing exosomal miRNA expression levels in ddPCR analysis. The application of endogenous reference with stable expression is beneficial in ddPCR data normalization, especially when the sample quality is highly variable. Furthermore, the expression stability of the four endogenous references were evaluated by ddPCR in 203 independent plasma samples, including 50 healthy volunteers, 34 CIN I patients, 56 CIN II-III patients, and 63 CC patients. The four inner control miRNAs showed invariable expression levels across these independent samples in both CIN I- and CIN II+ groups. The difference in expression of each of these inner control miRNAs was not significant between CIN I- and CIN II+ groups (*P* > 0.05) (Additional file 7: Figure S5), demonstrating the robustness and suitability of these inner controls in ddPCR analysis.

Then, the expression levels of the above three miRNAs (i.e., let-7a-3p, let-7d-3p, and miR-30d-5p) that were validated in tissues were quantified and normalized by ddPCR in 203 independent plasma samples. In our preliminary ddPCR analysis, let-7a-3p only produced about 2–10 copies /μl positive droplets using 4 ng input of exosomal miRNA sample, whereas the four inner controls and the other two miRNAs produced similar numbers of positive droplets with 0.02–0.1 ng input. Therefore, the less significantly expressed let-7a-3p in plasma exosomes was not pursued further in subsequent validations.

**Fig. 2:**
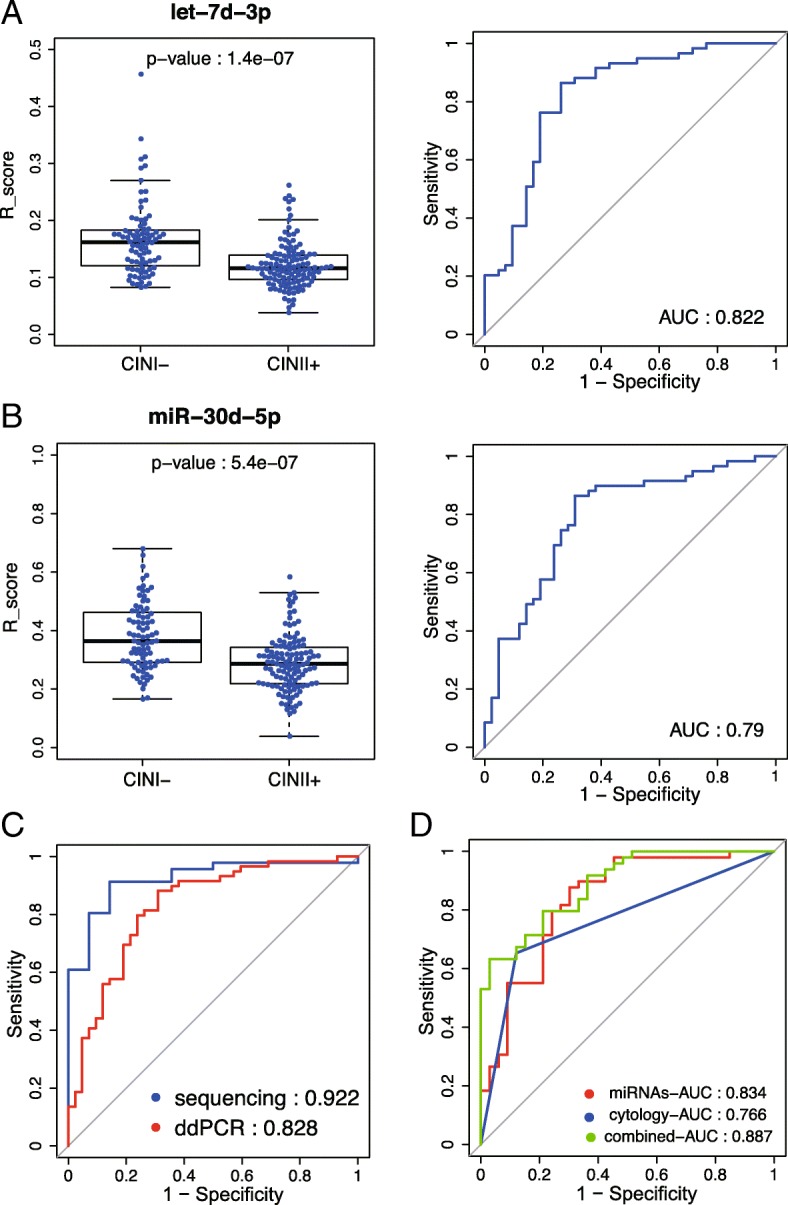
ddPCR validation of let-7d-3p and miR-30d-5p as diagnostic markers in 203 independent samples. **a** Expression and ROC analysis of let-7d-3p in validation samples (*P* = 1.4e-7 and AUC = 0.822). **b** Expression and ROC analysis of miR-30d-5p in validation samples (*P* = 5.4e-7 and AUC = 0.79). **c** ROC analysis of expression levels of two miRNAs (i.e., let-7d-5p and miR-30d-5p) from sequencing (AUC = 0.922, sequencing samples, *n* = 121) and ddPCR (AUC = 0.828, validation samples, *n* = 203). **d** ROC analysis of 166 validation samples that had at least one cytology test result. miRNA-AUC: ROC analysis based on two miRNAs (i.e., let-7d-5p and miR-30d-5p); cytology-AUC: ROC analysis based on cytology test results; combined-AUC: ROC analysis based on both miRNAs and cytology test results. All ROC analyses were performed to evaluate the sensitivity and specificity of exosomal miRNAs and/or cytology tests to discriminate CIN II+ from CIN I- subjects

The expression levels of let-7d-3p and miR-30d-5p was significantly decreased in the CIN II+ group when compared with the CIN I- group (Fig. 2a and b), which is consistent with both the plasma exosomal sequencing results and the qRT-PCR results of patients’ tissues as described above. The combination of the expression of let-7d-3p and miR-30d-5p from plasma exosomal sequencing in 121 training samples gave a distinguishing performance, with an AUC value of 0.922, whereas the AUC value based on the combined expression of these two DEmiRs from ddPCR in 203 validation plasma samples was 0.828 (Fig. 2c). A total of 166 of 203 validated samples had cytology test results. The AUC value based on cytology tests was 0.766, AUC value based on the miRNAs increased to 0.834, and integration of the two miRNAs in a cytological test-based model further achieved a higher AUC value of 0.887 (Fig. 2d). The positive predictive value and negative predictive value of the two-miRNA test were 0.95 and 0.75, respectively, in the discovery set, and the corresponding values were 0.80 and 0.81, respectively, in the validation set.

### Difference in miRNA expression profiles between circulation and tissues

To determine the spatial distribution and expression levels of miRNAs, we also measured seven miRNAs in the best panel (miR-320a, miR-128-3p, miR-129-5p, let-7d-3p, let-7i-5p, miR-30d-5p, and miR-30a-5p) in both plasma exosomes and in cancerous and adjacent normal tissues of CC patients using ddPCR analysis. There were different expression profiles between tissues and exosomes in the same validation system. The proportions of these miRNAs were, respectively, 57.5, 0.5, 2.9, 5.7, 9.1, 10.3, and 13.9% in tissues, and, respectively, 23.8, 22.8, 5.3, 7.5, 4.5, 18.2, and 17.9% in exosomes (Additional file 8: Figure S6). The Spearman correlation coefficient of miRNAs expression abundances between circulation and tissues was only 0.321 (*P* = 0.498), suggesting that exosomal miRNAs are likely selectively secreted from tumor cells. Although tumor cell content of sections from cervical tissue samples was confirmed by H&E staining to be > 70% in our study (Additional file 2: Supplementary Methods), these bulky tissue samples contained various amounts of stromal cells. It might be possible, although less likely, that exosomal miRNAs can be secreted by other cell types such as cancer-associated stromal cells in the tumor microenvironment.

## Conclusion

To our knowledge, the present study represents one of the largest plasma miRNA studies for cancer biomarker discovery. The identified exosomal miR-30d-5p and let-7d-3p are valuable diagnostic biomarkers for non-invasive screening of cervical cancer and its precursors. Blood extraction is more convenient and carries less risk of vaginal/uterine cervix infection than do TCT or Pap smear tests. It can also be incorporated into routine blood tests, which significantly reduces testing time for both patients and clinicians. Furthermore, these miRNAs in plasma exosomes are stable and resistant to physical degradation, making them promising diagnostic biomarkers for screening cervical cancer. However, further validation using large sample sizes is required before application in clinical diagnosis. Functional investigation of these miRNAs can give novel insights into their mechanism and physiologic relevance in the progression of cervical cancer.

## Additional file

Additional file 1: **Figure S1.** Flow chart for the study design. (PDF 217 kb)

Additional file 2: Supplementary Methods. (PDF 739 kb)

Additional file 3: **Figure S2.** Clinical summary of cytological detections (TCT and Pap smear) and biopsies in cervical cancer patients. (A) Number of patients in the study. Totally, 608 medical records of patients with cervical carcinoma and precancerous disease from 2015 to 2017 were collected. Among them, 456 had at least one TCT or Pap smear records, and 498 patients had tissue biopsy results. Ninety-eight of the 608 patients that had available peripheral blood samples were sequenced, among which 74 had at least one TCT or Pap smear record, and 89 had tissue biopsy results. The other 153 of the 608 patients that also had available peripheral blood samples were used as independent samples for ddPCR validation. Among these validation samples, 114 patients had at least one TCT or Pap smear record, and 127 patients had tissue biopsy results. The number of healthy volunteers for sequencing (23 subjects) and validation (50 subjects) were not presented in the summary Table. (B) The accuracy of cytological detections for all patients’ samples collected in the study. (C) The accuracy of biopsy results for all patients in the study. NILM: Negative for Intraepithelial Lesion or Malignancy; LSIL: Low-grade Squamous Intraepithelial Lesion; HSIL: High-grade Squamous Intraepithelial Lesion; ASC-US: Atypical Squamous Cell of Undetermined Significance; ASC-H: Atypical Squamous Cell-cannot exclude high-grade squamous intraepithelial lesion; AGC-NOS: Atypical Glandular Cells-not otherwise specified; CC: cervical cancer. NILM is regarded as normal, ASC/AGC is regarded as uncertain, LSIL is regarded as CIN I, HSIL is regarded as advanced CIN II and worse. For CIN I patients, the accuracy is defined as the proportion of CIN I that were identified as NILM or LSIL (i.e., CIN I-). For CIN II-III or CC patients, the accuracy is defined as the proportion of CIN II+ that were identified as HSIL, ASC-US, ASC-H or AGC-NOS. (PDF 497 kb)

Additional file 4: **Table S1.** miRNAs differentially expressed between CIN I- and CIN II+ groups in exosome sequencing data. (PDF 165 kb)

Additional file 5: **Figure S3.** Expression levels of the top eight significant miRNAs in tumor tissues and their adjacent tissues in cervical cancer patients. (A, B) qRT-PCR results of miRNAs in cervical cancer tissues. Green bars indicated that miRNAs were down-regulated in sequencing data (A), while yellow bars indicated that miRNAs were up-regulated in sequencing data (B). Fold change is defined as the ratio of miRNA expression in tumor to miRNA expression in adjacent normal tissue from the same patients. Forty-six new patients were analyzed in the study. (PDF 295 kb)

Additional file 6: **Figure S4.** Expression of four inner control miRNAs from next-generation sequencing data The gray lines with numbers represented ±95% confidence interval (CI) values. (PDF 327 kb)

Additional file 7: **Figure S5.** ddPCR results of four inner control miRNAs in 203 independent plasma samples. (PDF 227 kb)

Additional file 8: **Figure S6.** Distribution of seven miRNAs in tissue and exosome. (A) The average proportion of miRNAs in 46 paired tumor and adjacent normal tissues from cervical cancer patients. (B) The average proportion of miRNAs in 203 exosomes (84 samples from the CIN I- group and 119 samples from the CIN II+ group). The expression levels of miRNAs in both exosomal and tissue samples were measured by ddPCR. (PDF 273 kb)

## Abbreviations

ACC: Adenocarcinoma
AGC-NOS: Atypical Glandular Cells-not otherwise specified
ASC-H: Atypical Squamous Cell-cannot exclude high-grade squamous intraepithelial lesion
ASC-US: Atypical Squamous Cell of Undetermined Significance
AUC: Area under curve
CC: Cervical cancer
CIN: Cervical Intraepithelial Neoplasia
CV: Coefficient of variation
ddPCR: Droplet digital PCR
DEmiRs: Differentially expressed miRNAs
FDR: False discovery rate
HSIL: High-grade Squamous Intraepithelial Lesion
LSIL: Low-grade Squamous Intraepithelial Lesion
NILM: Negative for Intraepithelial Lesion or Malignancy
Pap smear: Papanicolaou test
PCA: Principal component analysis
ROC: Receiver operating characteristic
RPM: Reads Per Million mapped reads
SCC: Squamous cell carcinoma
TCT: ThinPrep Cytological Test
