# Supplementary figures and images for: Exosomal let-7d-3p and miR-30d-5p as diagnostic biomarkers for non-invasive screening of cervical cancer and its precursors
Source: Mol Cancer. 2019 Apr 2;18:76. doi: 10.1186/s12943-019-0999-x (PMC6446401; doi:10.1186/s12943-019-0999-x)

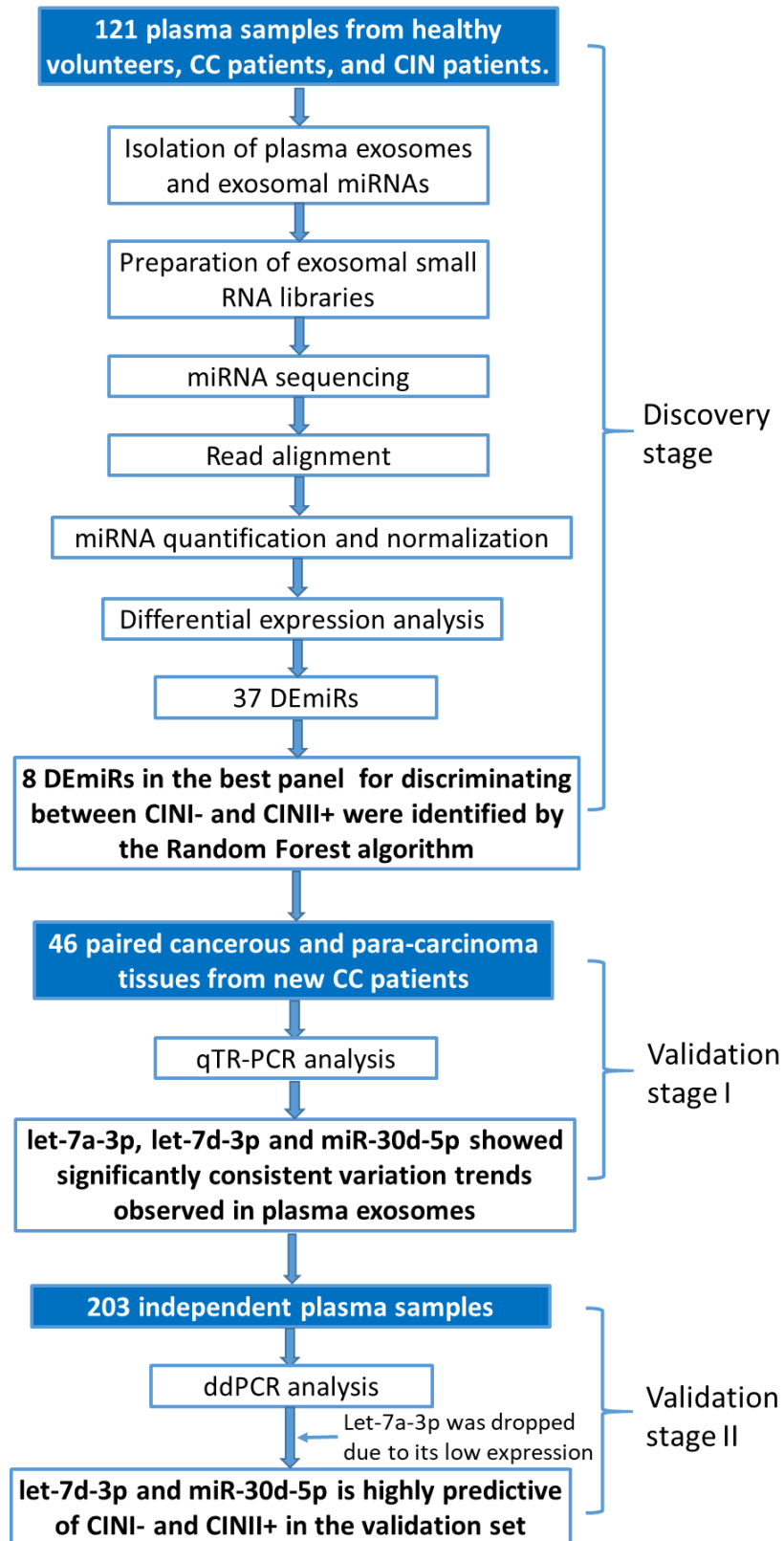

**Figure S1 Flow chart for the study design.**

Supplement: Supplementary file 1 — Figure S1. Flow chart for the study design. (PDF 217 kb) [file 12943_2019_999_MOESM1_ESM.pdf]

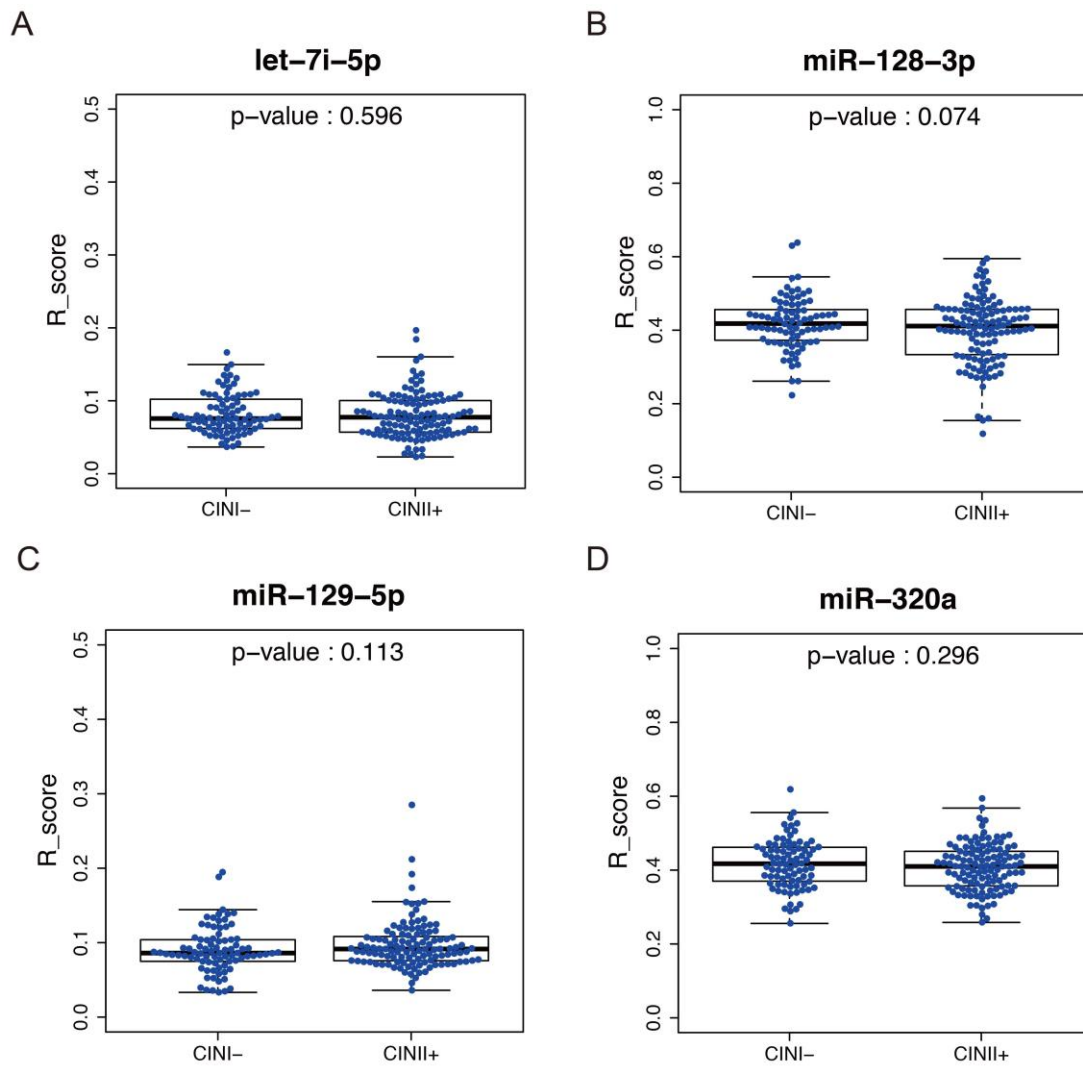

**Figure S5 ddPCR results of four inner control miRNAs in 203 independent plasma samples.**

Supplement: Supplementary file 7 — Figure S5. ddPCR results of four inner control miRNAs in 203 independent plasma samples. (PDF 227 kb) [file 12943_2019_999_MOESM7_ESM.pdf]
