# Supplementary material for: Exosomal let-7d-3p and miR-30d-5p as diagnostic biomarkers for non-invasive screening of cervical cancer and its precursors
Source: Mol Cancer. 2019 Apr 2;18:76. doi: 10.1186/s12943-019-0999-x (PMC6446401; doi:10.1186/s12943-019-0999-x)
Supplement: Supplementary file 2 — Supplementary Methods. (PDF 739 kb) [file 12943_2019_999_MOESM2_ESM.pdf]

Supplementary Methods

Patient sample collection

This study was reviewed and approved by the Ethnics Committees of Women's Hospital of Zhejiang University School of Medicine and Tongde Hospital of Zhejiang Province (Hangzhou, China). The study was conducted in accordance with the International Ethical Guidelines for Biomedical Research Involving Human Subjects. Medical records of 608 patients with cervical carcinoma and precancerous disease from 2015 to 2017 were reviewed to get clinical information, including age, Pap test results, TCT findings, pathology reports, and time of operation. Smoking status was not record in the study because the proportion of smoking in Chinese women is extremely low. The peripheral blood samples from 212 Cervical Intraepithelial Neoplasia II (CIN II) and higher (including CIN II, CIN III, and cervical carcinoma) patients and 112 healthy volunteers and Cervical Intraepithelial Neoplasia I (CIN I) patients were collected and centrifuged at 3,000 ×g for 10 min at 4°C to harvest plasma supernatant within 8 hours. Then the plasma samples were centrifuged at 16,000 ×g for 10 min at 4°C before storing at -80°C until use. The blood samples were only collected from patients who had not been treated with chemotherapy and radiotherapy before surgery. Patients with Loop Electrosurgical Excision Procedure surgery before total abdominal hysterectomy were only included in the study if there was pathological evidence with the presence of cancerous cells when the blood specimens were collected. Additional paired tumor and adjacent normal tissues from 46 cervical cancer patients were obtained at the time of diagnosis before any treatment was administered. Review of hematoxylin and eosin (H&E) slides was performed by a gynecologic pathologist to confirm the diagnosis. Tumor cell content of sections from frozen cervical tissue samples embedded in optimal cutting temperature medium was confirmed to be >70% by H&E staining. Fresh tissues were collected, snap-frozen in liquid nitrogen, and stored at -80°C.

Isolation of plasma exosomes and exosomal miRNAs

Plasma samples were centrifuged at 16,000 × g for 5 min at 4°C to remove possible residual cell debris after thawing the stored samples. The supernatants were incubated with pre-warmed thromboplastin D (Thermo Scientific Cat #: 100356, Grand Island, NY) for 15

min at 37°C. Exosomes were isolated using an ExoQuick Exosome Precipitation Solution (SBI Cat #:100356EXOQ20A-1, Mountain View, CA) mixture with RNase A (Sigma Cat#: R6513-10MG, St. Louis, MO) at a final concentration of 10 μl/ml to remove naked circulating RNA that might be co-precipitated along with exosomes at 4°C overnight. A total of 150 units/ml of murine RNase inhibitor (NEB Cat#: M0314L, Ipswich, MA) were added followed by precipitating the extracellular vesicles by centrifugation at 1500 × g for 5 min at room temperature. The exosome pellets were slightly washed and re-suspended in 25 μl PBS. The exosomal miRNA was isolated by miRNeasy Micro Kit (QIAGEN Cat#: 217084, Valencia, CA) following the manufacturer's protocol. The binding miRNA was then eluted with 14 μl of RNase-free water before storing at –80°C until use. The quality and quantity of the exosomal miRNA were measured by Small RNA Chip (Agilent Technologies Cat # 5067-1548, Santa Clara, CA) on the Agilent Bioanalyzer 2100 and using the Qubit™microRNA Assay Kit (Invitrogen Cat# Q32881) on the Qubit® 2.0 Fluorometer.

Preparation of exosomal small RNA library and sequencing

miRNA sequencing was performed for 121 plasma samples, including 23 healthy volunteers, 5 CIN I, 59 CIN II-III, 21 SCC and 13 adenocarcinoma (ACC) patients. Approximately 10 ng of small RNA from plasma exosomes were used to construct sequencing libraries using the NEBNext Multiplex Small RNA Library Prep Set (NEB Cat # E7560) following the manufacturer's protocol. The sequencing libraries were purified using the QIAquick PCR Purification Kit (QIAGEN Cat #: 28106) and run for size selection ranging from 140 to 160 bp by 3% agarose gel, then the miRNA libraries were acquired in 10 μl 10 mM Tris-HCI, pH 8.5. The size distribution and quantity of the libraries were evaluated using the DNA 1000 Kit (Agilent Technologies Cat #: 5067-1505) on an Agilent 2100 Bioanalyzer. Twenty-four libraries with different indexes were pooled at equal concentration before RNA sequencing. Pooled libraries were quantified by real-time qPCR using KAPA Library Quantification Kits (KAPA Biosystems, Wilmington, MA) and subjected to sequencing using an Illumina HiSeq X10 analyzer by WuXi Pharma Tech Company (Shanghai, China).

Sequencing data analysis

The adapter sequences were removed from the output short reads and sequences with low quality (base quality <20) at both ends of reads were further trimmed using Trim Galore (https://www.bioinformatics.babraham.ac.uk/projects/trim_galore/). The trimmed reads were aligned to the human reference genome (NCBI build 37) using the Burrows-Wheeler Aligner (1). Only one nucleotide mismatch was allowed in the mapping process. Read counts for miRNAs (miRBase v20) were extracted from alignment files using BEDtools (http://bedtools.readthedocs.io/en/latest). miRNA expression levels were quantified by Reads Per Million (RPM) mapped reads and then normalized with log2(RPM+1), which is the commonly used method for miRNAs quantification and normalization (2).

A permutation *t* test was performed to evaluate differential expression of exosomal miRNAs between different groups. The false discovery rate (FDR) was calculated to control error rates in multiple testing. The R package "gplots" was used to generate principal component analysis (PCA) plots and hierarchical clustering heatmaps on miRNA profiles. The R package "caret" was used to plot receiver operating characteristic (ROC) curves of miRNAs and calculate their area-under-curve (AUC) values. The ROC graph shows the diagnostic ability of a binary classifier system (e.g., CIN I- *versus* CIN II+) as its discrimination threshold is varied; the AUC value ranges between [0, 1]. A value of 1 represents perfect performance and a value of 0.5 represents random performance. The R package "Random Forest" was used to select feature vectors that formed the best panel to discriminate two different groups using the machine learning function. All statistical analyses and graphics were implemented in R statistical packages (www.r-project.org).

Quantitative PCR validation in cervical cancer tissues

Total RNA was extracted from 20–30 mg of tumor and adjacent normal tissues by TRIzol (Life Technologies Cat#: 15596018). The quality and quantity of RNA were determined by NanoDrop ND-1000 UV-Vis Spectrophotometer (Thermo Fisher Scientific, Waltham, MA). A total of 500 ng of total RNA were reverse transcribed using miRNA first-strand cDNA synthesis (tailing reaction) Kit (Sangon Biotech, China Cat#: B532451) following the manufacturer's protocol. PCR reaction was performed in triplicate using SYBR®Premix Ex Taq™Kit (Takara Cat#: RR420A) and ViiA™7 Real-time PCR System.

Primers of miRNAs were synthesized with specific customization by Sangon Biotech, China (**Additional file 1**, **Table S1**). Universal internal control U6 snRNA (Sangon Biotech, China) was used in the PCR validation.

miRNA primers in validation experiments.

miRNA	Primer Sequencing
let-7a-3p	cgcgcgctatacaatctactgtctttc
let-7d-3p	gctatacgacctgctgcctttct
miR-144-5p	ccgcgcgggatatcatcatatactgtaag
miR-182-5p	cgtttggcaatggtagaactcacact
miR-183-5p	gcgtatggcactggtagaattcact
miR-215-5p	gcgcgatgacctatgaattgacagac
miR-30a-5p	gcgtgtaaacatcctcgactggaag
miR-30d-5p	cgtgtaaacatccccgactggaag
miR-4443	gcttggaggcgtgggtttt
let-7i-5p	gcgtgaggtagtagtttgtgctgtt
miR-128-3p	cgtcacagtgaaccggtctcttt
miR-129-5p	ctttttgcggtctgggcttgc
miR-320a	aaaagctgggttgagagggcg

Identification of endogenous references for exosomal miRNAs

The normalized miRNA expression data were first screened for the most stably expressed miRNAs with an overall coefficient of variation (CV) <5%. As a result, four relatively stable high-expression miRNAs (miR-128-3p, miR-129-5p, miR-320a, and Let-7i-5p) were chosen as internal controls in ddPCR validation in exosomes. Their scatter diagrams of log2(RPM+1) were almost within their ±95% confidence intervals (**Additional file 2**, **Fig. S1**). Their relative log2(PRM+1) values were, respectively, 14.41±0.64, 15.46±0.47, 12.88±0.39, and 12.83±0.51, and their CVs were, respectively, 4.44%, 3.02%, 3.04%, and 3.97%. The ddPCR uses absolute quantitative principles, so the following formula was used to normalize every miRNA expression:

$$\mathrm{R\_score} = \frac{\mathrm{Exp\_test}}{\sum_{i=1}^{4} \mathrm{Exp\_con}}$$

where R_score represents the ratio of expression level of an miRNA to the sum of the miRNAs expression levels of four inner controls in a validation sample; Exp_test represents the absolute expression of each tested miRNA; and Exp_con represents the absolute expression of the four inner controls.

ddPCR validation in independent samples

Each plasma exosomal miRNA sample was diluted to 0.02 ng/μl. The expression levels of plasma exosomal miRNAs were evaluated by the QX200 ddPCR system (BioRad) with EvaGreen Supermix (Biorad Cat#: 186-4035) (3). Exactly 20 μl of each ddPCR assay mixture were loaded into a disposable droplet generator cartridge with 70 ul of droplet-generation oil for EvaGreen, and about 40 ul of droplets were then produced by the QX200 droplet generator. Thermal cycling conditions for EvaGreen assays were as follows: 95°C for 5 minutes, then 40 cycles of 95°C for 30 seconds and 60°C for 1 minute, and two final steps at 4°C for 5 minutes, 90°C for 10 minutes (all ramping rate reduced to 2°C/s), and a 4°C indefinite hold to enhance dye stabilization (3). The P value was calculated by two-sided *t* tests to compare miRNA expression between groups. Similarly, the R package "caret" was used to draw ROC curves and calculate AUC values.

Target genes and pathway analysis of the validated miRNAs

In silico analysis to identify miRNA target genes and pathways was done by DIANA tools. Experimentally validated evidence for miRNAs and their target genes were extracted from TarBase (4). Pathway enrichment analysis of the validated miRNAs was performed by DIANA-miRPath (5). The significance of the Gene Ontology category and KEGG pathway enrichment were tested using the two-sided Fisher's exact test and the Chi-squared test. FDR < 0.01 was used to select significant Gene Ontology categories and KEGG pathways (6). Cytoscape (7) was used to draw the connection network of genes interacting with at least three miRNAs.

References:

1. Li H, Durbin R. Fast and accurate short read alignment with Burrows-Wheeler transform. Bioinformatics **2009**;25(14):1754-60 doi 10.1093/bioinformatics/btp324.

2. Fan JB. Next-Generation MicroRNA Expression Profiling Technology. Methods in Molecular Biology **2012**;822.
3. Miotto E, Saccenti E, Lupini L, Callegari E, Negrini M, Ferracin M. Quantification of circulating miRNAs by droplet digital PCR: comparison of EvaGreen- and TaqMan-based chemistries. Cancer Epidemiol Biomarkers Prev **2014**;23(12):2638-42 doi 10.1158/1055-9965.EPI-14-0503.
4. Reczko M, Maragkakis M, Alexiou P, Grosse I, Hatzigeorgiou AG. Functional microRNA targets in protein coding sequences. Bioinformatics **2012**;28(6):771-6 doi 10.1093/bioinformatics/bts043.
5. Vlachos IS, Paraskevopoulou MD, Karagkouni D, Georgakilas G, Vergoulis T, Kanellos I*, et al.* DIANA-TarBase v7.0: indexing more than half a million experimentally supported miRNA:mRNA interactions. Nucleic Acids Research **2015**;43(D1):D153-D9 doi 10.1093/nar/gku1215.
6. Alhasan AH, Scott AW, Wu JJ, Feng G, Meeks JJ, Thaxton CS*, et al.* Circulating microRNA signature for the diagnosis of very high-risk prostate cancer. Proc Natl Acad Sci U S A **2016**;113(38):10655-60 doi 10.1073/pnas.1611596113.
7. Shannon P, Markiel A, Ozier O, Baliga NS, Wang JT, Ramage D*, et al.* Cytoscape: a software environment for integrated models of biomolecular interaction networks. Genome Res **2003**;13(11):2498-504 doi 10.1101/gr.1239303.
