# Supplementary material for: Exosomal let-7d-3p and miR-30d-5p as diagnostic biomarkers for non-invasive screening of cervical cancer and its precursors
Source: Mol Cancer. 2019 Apr 2;18:76. doi: 10.1186/s12943-019-0999-x (PMC6446401; doi:10.1186/s12943-019-0999-x)
Supplement: Supplementary file 3 — Figure S2. Clinical summary of cytological detections (TCT and Pap smear) and biopsies in cervical cancer patients. (A) Number of patients in the study. Totally, 608 medical records of patients with cervical carcinoma and precancerous disease from 2015 to 2017 were collected. Among them, 456 had at least one TCT or Pap smear records, and 498 patients had tissue biopsy results. Ninety-eight of the 608 patients that had available peripheral blood samples were sequenced, among which 74 had at least one TCT or Pap smear record, and 89 had tissue biopsy results. The other 153 of the 608 patients that also had available peripheral blood samples were used as independent samples for ddPCR validation. Among these validation samples, 114 patients had at least one TCT or Pap smear record, and 127 patients had tissue biopsy results. The number of healthy volunteers for sequencing (23 subjects) and validation (50 subjects) were not presented in the summary Table. (B) The accuracy of cytological detections for all patients’ samples collected in the study. (C) The accuracy of biopsy results for all patients in the study. NILM: Negative for Intraepithelial Lesion or Malignancy; LSIL: Low-grade Squamous Intraepithelial Lesion; HSIL: High-grade Squamous Intraepithelial Lesion; ASC-US: Atypical Squamous Cell of Undetermined Significance; ASC-H: Atypical Squamous Cell-cannot exclude high-grade squamous intraepithelial lesion; AGC-NOS: Atypical Glandular Cells-not otherwise specified; CC: cervical cancer. NILM is regarded as normal, ASC/AGC is regarded as uncertain, LSIL is regarded as CIN I, HSIL is regarded as advanced CIN II and worse. For CIN I patients, the accuracy is defined as the proportion of CIN I that were identified as NILM or LSIL (i.e., CIN I-). For CIN II-III or CC patients, the accuracy is defined as the proportion of CIN II+ that were identified as HSIL, ASC-US, ASC-H or AGC-NOS. (PDF 497 kb) [file 12943_2019_999_MOESM3_ESM.pdf]

A

Samples	Total Patients					Sequencing Patients					Validated Patients				
**Cytological Result***	**CINI**	**CINII-III**	**ACC**	**SCC**	**total**	**CINI**	**CINII-III**	**ACC**	**SCC**	**total**	**CINI**	**CINII-III**	**ACC**	**SCC**	**total**
**HSIL**	0	59	4	64	127	0	11	1	5	17	0	15	0	11	26
**LSIL**	12	20	1	10	43	1	4	0	1	6	4	2	0	6	12
**AGC/ASC**	11	80	19	65	175	0	18	4	5	27	4	20	3	18	45
**NILM**	11	53	11	36	111	1	13	8	2	24	5	15	1	10	31
**total**	34	212	35	175	456	2	46	13	13	74	13	52	4	45	114
**Biopsy Result**	**CINI**	**CINII-III**	**ACC**	**SCC**	**total**	**CINI**	**CINII-III**	**ACC**	**SCC**	**total**	**CINI**	**CINII-III**	**ACC**	**SCC**	**total**
**HSIL**	9	205	35	194	443	0	43	13	24	80	3	51	6	51	111
**LSIL**	30	14	2	5	51	4	3	1	0	8	11	2	0	2	15
**NILM**	0	2	0	2	4	0	1	0	0	1	0	0	0	1	1
**total**	39	221	37	201	498	4	47	14	24	89	14	53	6	54	127

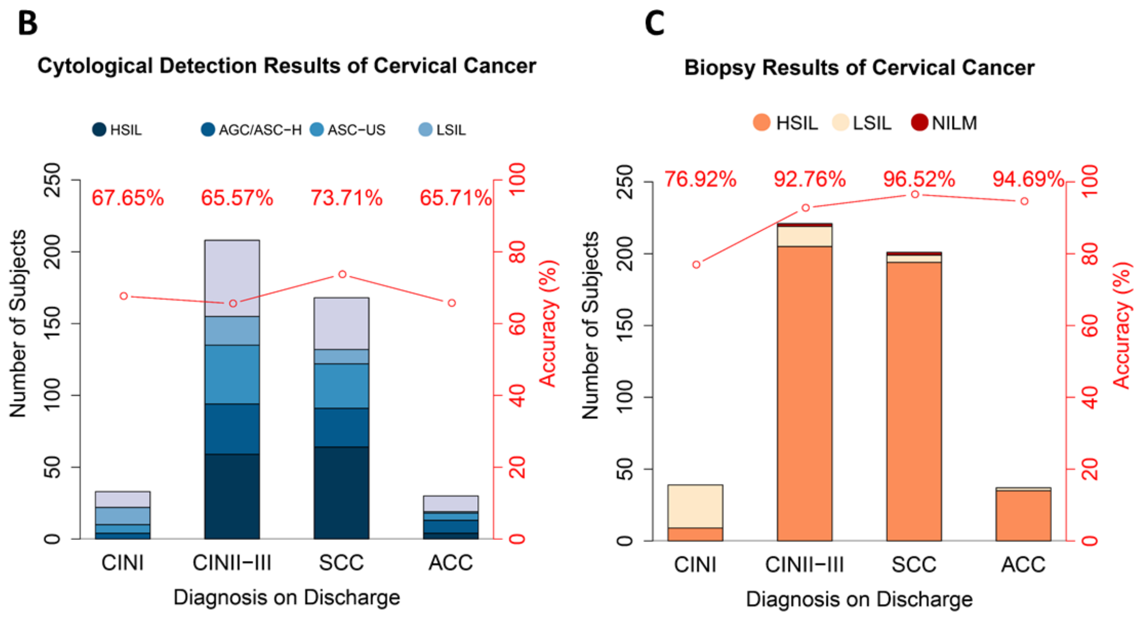

Figure S2 Clinical summary of cytological detections (TCT and Pap smear) and biopsies in cervical cancer patients

(**A**) Number of patients in the study. Totally, 608 medical records of patients with cervical carcinoma and precancerous disease from 2015 to 2017 were collected. Among them, 456 had at least one TCT or Pap smear records, and 498 patients had tissue biopsy results. Ninety-

eight of the 608 patients that had available peripheral blood samples were sequenced, among which 74 had at least one TCT or Pap smear record, and 89 had tissue biopsy results. The other 153 of the 608 patients that also had available peripheral blood samples were used as independent samples for ddPCR validation. Among these validation samples, 114 patients had at least one TCT or Pap smear record, and 127 patients had tissue biopsy results. The number of healthy volunteers for sequencing (23 subjects) and validation (50 subjects) were not presented in the summary table. (**B**) The accuracy of cytological detections for all patients' samples collected in the study. (**C**) The accuracy of biopsy results for all patients in the study. NILM: Negative for Intraepithelial Lesion or Malignancy; LSIL: Low-grade Squamous Intraepithelial Lesion; HSIL: High-grade Squamous Intraepithelial Lesion; ASC-US: Atypical Squamous Cell of Undetermined Significance; ASC-H: Atypical Squamous Cell-cannot exclude high-grade squamous intraepithelial lesion; AGC-NOS: Atypical Glandular Cells-not otherwise specified; CC: cervical cancer. NILM is regarded as normal, ASC/AGC is regarded as uncertain, LSIL is regarded as CIN I, HSIL is regarded as advanced CIN II and worse. For CIN I patients, the accuracy is defined as the proportion of CIN I that were identified as NILM or LSIL (i.e., CIN I-). For CIN II-III or CC patients, the accuracy is defined as the proportion of CIN II+ that were identified as HSIL, ASC-US, ASC-H or AGC-NOS.
