# Supplementary material for: Exosomal let-7d-3p and miR-30d-5p as diagnostic biomarkers for non-invasive screening of cervical cancer and its precursors
Source: Mol Cancer. 2019 Apr 2;18:76. doi: 10.1186/s12943-019-0999-x (PMC6446401; doi:10.1186/s12943-019-0999-x)
Supplement: Supplementary file 4 — Table S1. miRNAs differentially expressed between CIN I- and CIN II+ groups in exosome sequencing data. (PDF 165 kb) [file 12943_2019_999_MOESM4_ESM.pdf]

Table S1 miRNAs differentially expressed between CIN I- and CIN II+ groups in exosome sequencing data.

miRNAs	mean+/-SE (CIN I-)	mean+/-SE (CIN II+)	pvalue.log2	pvalue.qq	log2.fdr	qq.fdr
hsa-miR-144-5p	1.14+/-0.40	2.19+/-0.86	1.01E-15	7.73E-20	3.58E-12	1.54E-10
hsa-miR-4443	0.95+/-0.23	1.49+/-0.41	9.59E-14	1.33E-12	2.34E-08	3.38E-08
hsa-let-7d-3p	6.37+/-0.44	5.43+/-0.71	2.60E-14	1.19E-09	1.00E-11	7.24E-08
hsa-let-7a-3p	5.72+/-0.48	4.98+/-0.62	8.34E-09	2.94E-10	1.18E-06	7.24E-08
hsa-miR-183-5p	3.74+/-0.33	4.32+/-0.63	3.76E-09	1.55E-09	6.56E-07	3.15E-07
hsa-miR-30a-5p	9.94+/-0.37	9.47+/-0.42	1.23E-06	4.27E-09	3.05E-05	4.90E-07
hsa-miR-96-5p	0.86+/-0.36	1.21+/-0.52	0.000185	1.04E-08	0.003373	4.90E-07
hsa-miR-574-3p	1.59+/-0.42	2.06+/-0.71	2.55E-05	1.27E-08	0.001765	3.19E-06
hsa-miR-30d-5p	9.06+/-0.49	8.25+/-0.54	1.63E-10	1.11E-07	5.15E-07	3.24E-06
hsa-miR-182-5p	4.02+/-0.55	4.80+/-0.72	4.33E-08	4.03E-08	2.97E-06	3.24E-06
hsa-miR-337-3p	1.35+/-0.29	0.94+/-0.34	1.66E-08	1.21E-07	2.97E-06	3.24E-06
hsa-miR-148b-3p	8.93+/-0.50	8.20+/-0.57	5.80E-08	1.24E-07	6.48E-06	3.04E-05
hsa-let-7b-3p	4.22+/-0.37	3.78+/-0.59	4.27E-06	8.17E-07	0.000354	3.04E-05
hsa-miR-181b-5p	11.55+/-0.39	11.09+/-0.66	2.25E-05	6.30E-07	0.000582	3.04E-05
hsa-miR-181a-5p	11.85+/-0.41	11.32+/-0.64	8.52E-07	1.62E-06	3.05E-05	6.14E-05
hsa-miR-656-3p	8.74+/-0.60	8.00+/-0.91	2.80E-06	9.13E-07	0.000105	6.14E-05
hsa-miR-215-5p	2.23+/-0.55	1.46+/-0.71	1.03E-07	4.11E-06	1.57E-05	0.000118
hsa-miR-136-5p	2.49+/-0.46	1.88+/-0.71	9.04E-07	1.97E-06	3.05E-05	0.000118
hsa-let-7f-1-3p	4.02+/-0.45	3.52+/-0.51	7.17E-06	7.60E-06	0.000354	0.000118
hsa-miR-409-5p	3.62+/-0.36	3.25+/-0.41	1.61E-05	6.93E-06	0.000582	0.000118
hsa-miR-99a-5p	15.98+/-0.35	15.63+/-0.52	2.04E-05	8.62E-06	0.000582	0.000118
hsa-miR-27b-3p	11.49+/-0.45	11.07+/-0.56	0.00032	8.07E-06	0.006445	0.000118
hsa-miR-455-5p	4.90+/-0.46	4.38+/-0.50	4.07E-06	9.10E-06	0.000105	0.000579
hsa-miR-483-5p	2.40+/-0.30	2.86+/-0.79	9.21E-06	2.10E-05	0.000582	0.000579
hsa-miR-885-5p	2.92+/-0.53	2.48+/-0.69	0.000311	2.41E-05	0.003373	0.000946
hsa-miR-320e	2.49+/-0.31	2.83+/-0.47	1.99E-06	3.23E-05	5.44E-05	0.001581
hsa-miR-10b-5p	7.89+/-0.46	7.27+/-0.56	2.13E-07	0.000258	3.05E-05	0.002798
hsa-miR-1468-5p	1.40+/-0.37	0.96+/-0.36	5.26E-07	0.000214	3.05E-05	0.002798
hsa-miR-105-5p	6.88+/-0.46	6.37+/-0.56	2.77E-06	0.000164	5.44E-05	0.002798
hsa-miR-339-5p	2.88+/-0.53	2.42+/-0.59	0.000154	0.000361	0.003373	0.002798
hsa-miR-128-3p	14.69+/-0.46	14.32+/-0.67	0.000245	0.000338	0.003373	0.002798
hsa-miR-381-3p	12.42+/-0.54	12.82+/-0.57	0.000403	0.000248	0.006445	0.002798
hsa-miR-432-5p	8.82+/-0.47	8.43+/-0.45	0.00041	9.97E-05	0.006953	0.002798
hsa-miR-543	6.94+/-0.52	6.41+/-0.71	2.23E-06	0.000803	5.44E-05	0.006533
hsa-miR-365a-3p	1.34+/-0.42	1.00+/-0.39	0.000267	0.001077	0.003373	0.006533
hsa-miR-145-3p	5.79+/-0.49	5.33+/-0.62	0.00018	0.000688	0.003373	0.006533
hsa-miR-889-3p	7.12+/-0.66	6.52+/-0.65	0.000173	0.001081	0.003373	0.006533

Note: RPM was transformed with either log2 or quantile-quantile before the analysis. SE: standard error.
