# Supplementary material for: Exosomal let-7d-3p and miR-30d-5p as diagnostic biomarkers for non-invasive screening of cervical cancer and its precursors
Source: Mol Cancer. 2019 Apr 2;18:76. doi: 10.1186/s12943-019-0999-x (PMC6446401; doi:10.1186/s12943-019-0999-x)
Supplement: Supplementary file 5 — Figure S3. Expression levels of the top eight significant miRNAs in tumor tissues and their adjacent tissues in cervical cancer patients. (A, B) qRT-PCR results of miRNAs in cervical cancer tissues. Green bars indicated that miRNAs were down-regulated in sequencing data (A), while yellow bars indicated that miRNAs were up-regulated in sequencing data (B). Fold change is defined as the ratio of miRNA expression in tumor to miRNA expression in adjacent normal tissue from the same patients. Forty-six new patients were analyzed in the study. (PDF 295 kb) [file 12943_2019_999_MOESM5_ESM.pdf]

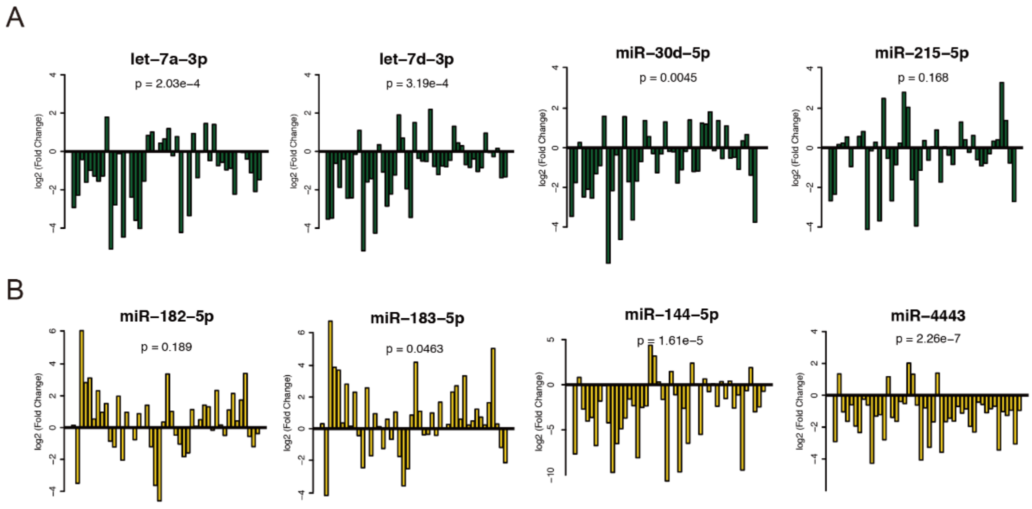

Figure S3 Expression levels of the top eight significant miRNAs in tumor tissues and their adjacent tissues in cervical cancer patients

(**A**, **B**) qRT-PCR results of miRNAs in cervical cancer tissues. Green bars indicated that miRNAs were down-regulated in sequencing data (**A**), while yellow bars indicated that miRNAs were up-regulated in sequencing data (**B**). Fold change is defined as the ratio of miRNA expression in tumor to miRNA expression in adjacent normal tissue from the same patients. Forty-six new patients were analyzed in the study.
