# Supplementary material for: Exosomal let-7d-3p and miR-30d-5p as diagnostic biomarkers for non-invasive screening of cervical cancer and its precursors
Source: Mol Cancer. 2019 Apr 2;18:76. doi: 10.1186/s12943-019-0999-x (PMC6446401; doi:10.1186/s12943-019-0999-x)
Supplement: Supplementary file 6 — Figure S4. Expression of four inner control miRNAs from next-generation sequencing data The gray lines with numbers represented ±95% confidence interval (CI) values. (PDF 327 kb) [file 12943_2019_999_MOESM6_ESM.pdf]

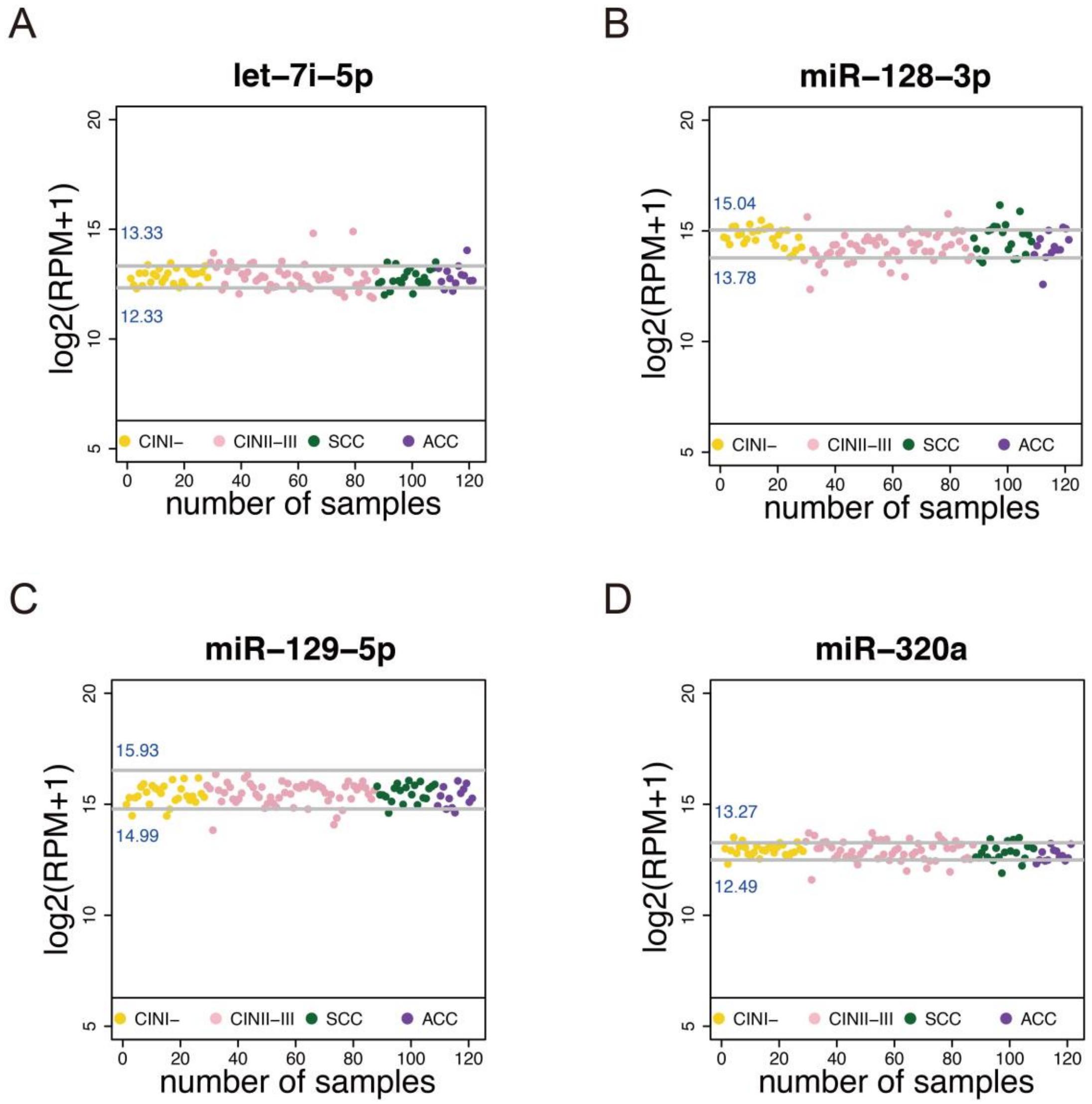

Figure S4 Expression of four inner control miRNAs from next-generation sequencing data The gray lines with numbers represented ±95% confidence interval (CI) values.
