# Supplementary material for: Exosomal let-7d-3p and miR-30d-5p as diagnostic biomarkers for non-invasive screening of cervical cancer and its precursors
Source: Mol Cancer. 2019 Apr 2;18:76. doi: 10.1186/s12943-019-0999-x (PMC6446401; doi:10.1186/s12943-019-0999-x)
Supplement: Supplementary file 8 — Figure S6. Distribution of seven miRNAs in tissue and exosome. (A) The average proportion of miRNAs in 46 paired tumor and adjacent normal tissues from cervical cancer patients. (B) The average proportion of miRNAs in 203 exosomes (84 samples from the CIN I- group and 119 samples from the CIN II+ group). The expression levels of miRNAs in both exosomal and tissue samples were measured by ddPCR. (PDF 273 kb) [file 12943_2019_999_MOESM8_ESM.pdf]

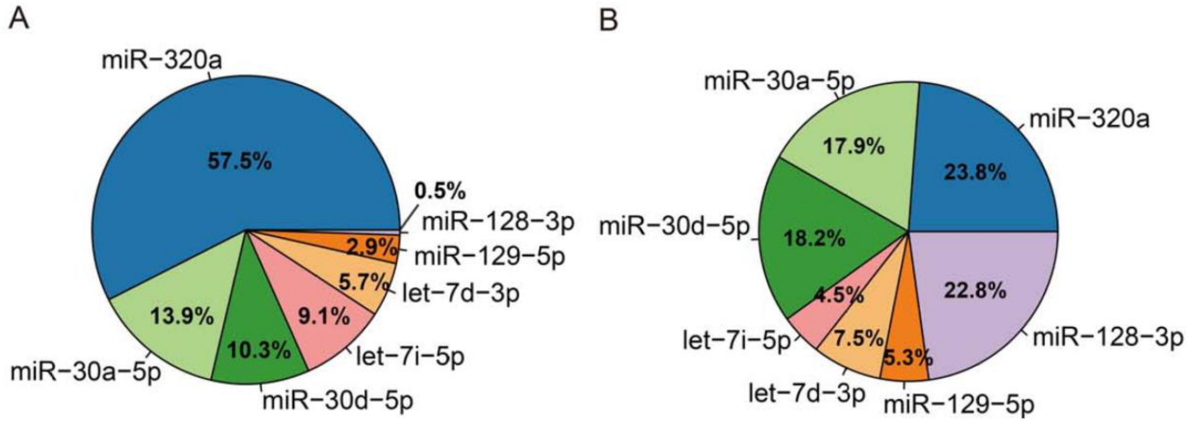

Figure S6 Distribution of seven miRNAs in tissue and exosome

(**A**) The average proportion of miRNAs in 46 paired tumor and adjacent normal tissues from cervical cancer patients. (**B**) The average proportion of miRNAs in 203 exosomes (84 samples from the CIN I- group and 119 samples from the CIN II+ group). The expression levels of miRNAs in both exosomal and tissue samples were measured by ddPCR.
